# Peer support to improve the secondary distribution of Internet-based HIV self-testing kits among men who have sex with men in Zhuhai, China

**DOI:** 10.3389/fpubh.2025.1522425

**Published:** 2025-04-29

**Authors:** Hang Lyu, Yuxin Ni, Xi He, Dongya Wang, Xinxue Yu, Weiming Tang, Yi Zhou

**Affiliations:** ^1^Zhuhai Center for Disease Control and Prevention, Zhuhai, Guangdong, China; ^2^University of North Carolina Project-China, Guangzhou, China; ^3^Department of Health Law, Policy and Management, Boston University School of Public Health, Boston, MA, United States; ^4^Zhuhai Xutong Voluntary Services Center, Zhuhai, China; ^5^Department of Strategic Communication, University of Miami, Coral Gables, FL, United States; ^6^Department of Public Health and Preventive Medicine, School of Medicine, Jinan University, Guangzhou, China; ^7^Dermatology Hospital of South Medical University, Guangzhou, China; ^8^Faculty of Medicine, Macau University of Science and Technology, Macau, Macao SAR, China

**Keywords:** men who have sex with men, HIV, HIV self-testing, peer support, behavior

## Abstract

**Background:**

Support from peers is a commonly employed behavioral prevention approach aimed at key populations to enhance HIV prevention efforts. Internet-based HIV self-testing (HIVST) has been widely used among men who have sex with men (MSM) across China over the past few years.

**Objective:**

We aimed to analyze the occurrence of Internet-based peer support and high-risk behaviors during the process of distributing HIVST kits among MSM.

**Methods:**

The single-arm prospective cohort study was conducted among MSM in Zhuhai, China. The study utilized an HIVST online ordering system based on WeChat, which was developed by Xutong. MSM who ordered HIVST kits and distributed them to people in their network for self-testing were defined as index participants. People who received the kits from the index participants and provided testing results were defined as alters. Chi-squared tests were used to compare baseline and follow-up characteristics of the participants. Cramer’s V was used to quantify the level of association between the Internet-based HIVST and condomless anal sexual behaviors among index participants. Logistic regression analysis was used to evaluate factors associated with peer support among alter participants. A *p*-value <0.05 was regarded as statistically significant.

**Results:**

A total of 288 index participants were included in the study, and they distributed HIVST kits to 478 alters. In the study, 84.0% (242/288) of the index participants reported that they provided peer support to their alters, and 87.4% (418/478) of the alters reported receiving peer support from the index participants. As for the alters, 19.9% (95/478) had sex with index participants on the day of HIVST. Among them, 24.2% (23/95) had sex after the HIVST and 72.6% (69/95) used a condom during sex. Index participants who had been asked for advice about HIV and other sexually transmitted infections were more likely to provide peer support to alters (OR: 1.85, 95% CI: 1.01–3.37).

**Conclusion:**

This study has enhanced our understanding of the occurrence of peer support and high-risk behaviors during the process of HIVST kits secondary distribution and provided evidence that Internet-based HIVST appears to be a promising approach for behaviors interventions among MSM.

## Introduction

In China, sexual transmission is the primary route of human immunodeficiency virus (HIV) transmission (1). A report by the Chinese Ministry of Health estimated that approximately 1.05 million people were living with HIV/AIDS in China in 2020, and 23.3% of the newly identified HIV/AIDS cases were attributable to male-to-male sexual contact (1, 2). Behavioral prevention remains central to the effort to reduce HIV transmission. Peer-supported intervention is a widely used behavioral prevention strategy targeting key populations to promote HIV prevention (3–5). A peer-based intervention program conducted among MSM in China has proven that peer support can be used to improve HIV testing and linkage to care (6). A meta-analysis of 15 studies also found that peer-led HIV interventions might reduce overall unprotected anal intercourse among HIV-negative MSM (7). Another meta-analysis published on JMIR recently also found that Internet/digital-based interventions are now most commonly used to promote and facilitate HIV testing among MSM (8). A program carried out in Brazil has also proven that E-testing proved highly feasible and acceptable, supporting scale-up to additional centers for MSM (9). Support for the use of peers in a wide variety of HIV care settings is likely based on their perceived availability and accessibility (10).

HIV self-testing (HIVST) has been shown as a cost-effective approach to supplement HIV testing services and reach individuals who have avoided facility-based testing (11). A systematic review and meta-analysis conducted in China showed that HIVST has become a valuable tool for HIV prevention in China and is widely spread (12). A national cross-sectional study in China has proven that distributing HIVST application links among the MSM population via Internet-based social media is feasible (13). A previous study by our team showed that peer-based secondary distribution of HIVST kits holds promise to increase HIV testing coverage and case identification among MSM (14). HIVST secondary distribution encourages MSM to apply multiple HIVST kits (index) and distribute them to people within their social networks, including sexual partners and friends (alters) (15). A program data analysis carried out in Côte d’Ivoire, West Africa, showed that enhanced peer outreach was effective, even in reaching female sex workers for HIV prevention and treatment (16). As a result, peers have access to a broader network of MSM than researchers and traditional medical clinics.

In addition, counseling and information provided through HIV testing services may provide opportunities to influence subsequent sexual behaviors, particularly in terms of reducing the number of sexual partners and increasing condom-protected sex among HIV-positive individuals. This, in turn, can impact HIV transmission and prevention (17–19). These changes in behaviors may be attributable to supportive information provided at the time of HIV testing, testing behaviors, or knowledge of HIV serostatus. However, few studies have assessed whether the individuals (referred to as “alters”) received peer support (i.e., messages on HIV, what HIVST is, and how to conduct HIVST) during the process of HIVST distribution by index participants; a big part of whom never tested for HIV before and may have higher-risk behaviors compared to other MSM.

Therefore, this study aimed to analyze the occurrence of peer support and high-risk behaviors between index participants and alters during the process of HIVST kits distribution.

## Methods

### Study design and participants

This single-arm prospective cohort study was carried out in Zhuhai, a city located in southern China, where an estimated 17,000 MSM reside. This study was conducted jointly by the University of North Carolina at Chapel Hill Project-China, Zhuhai Center for Diseases Control and Prevention (CDC), and Zhuhai Xutong Voluntary Services Center (Xutong). Xutong is a gay-friendly community-based organization in Zhuhai, providing HIV prevention services (e.g., condoms, testing, and linkage to treatment) to men who have had sex with men for many years. The study utilized an HIVST online ordering system developed by Xutong. The ordering system was hosted and managed using WeChat, China’s largest social networking platform. Based on the pilot study’s results and the characteristics of single-arm prospective cohort studies, we set the index’s transmission proportion at 1.2, with an alpha level of 0.05, a power of 0.90, and a 20% dropout rate. We estimated the sample size for the index group to be 208 individuals of index participants and 249 for alters. In this study, we collected data from two groups of population: (1) the Index group, who applied HIVST kits and distributed them to people within their social networks, including sexual partners and friends; (2) the Alter group, who received HIVST kits from the index participants. To observe the peer support, we only selected all index participants who distributed HIVST and alters who received HIVST from one of the index participants and provided feedback on testing results. Index participants did not receive any training on how to distribute HIVST.

The inclusion criteria of the index participants were as follows: (1) assigned male at birth; (2) aged 18 years or older; (3) ever had male-to-male sex; (4) ordered HIVST kits through the online platform; and (5) be consented to take baseline and follow-up surveys online. All index participants and alters agreed to sign informed consent forms before the online baseline survey. The inclusion criteria of the alter participants were as follows: (1) assigned male at birth; (2) aged 18 years or older; (3) received HIVST kits through index; and (4) consented to take follow-up surveys online.

### Procedures

**Figure 1:**
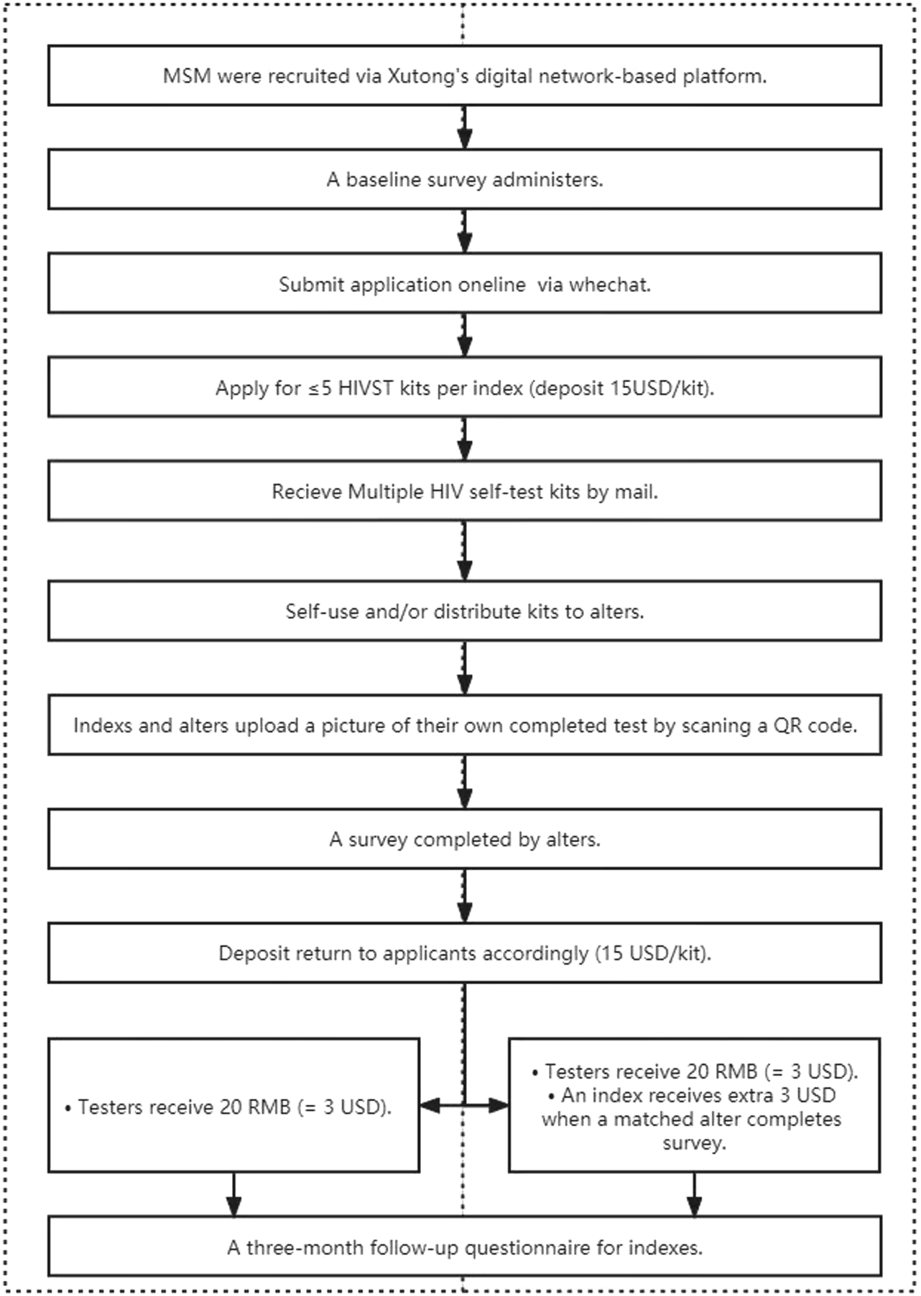
Study flow diagram of the internet-based HIVST kits secondary distribution among MSM in Zhuhai, China.

MSM were able to apply for HIVST kits for free using Xutong’s public WeChat account. HIVST kits were mailed to index men after they paid the deposit (US$15 per kit) and provided shipping information. Upon receiving a photographed test result and verified by a Xutong staff member trained in HIV testing, the deposit was immediately refunded to index men through WeChat. The index group completed the baseline survey and ordered HIVST kits (SD Bioline, South Korea) through the online system. The SD BIOLINE HIV/syphilis duo test kits (Standard Diagnostics Inc.) were utilized in this study. Each index participant could conduct self-tests or distribute the HIVST kits to alters. After testing, all testers scanned the QR code in a returning card in each HIVST kit to submit photographs of their test results and reported whether they were HIVST kit index participants or alters. Alters completed and submitted the baseline survey when they received HIVST kits from index participants and subsequently submitted their test results. In the second phase of the study, we used an additional monetary incentive to increase the number of HIVST kit applications and distribution from the index participants. In this phase, in addition to the fixed monetary incentive, both the index participants who distributed HIVST to the alter and the alters who returned the test results received an additional 20 RMB (≈3 USD) as monetary incentive. Individuals with positive test results for HIV were referred for confirmatory testing at the local CDC and provided other counseling and linkage to care services by volunteers. Trained staff of Xutong ensured the confidentiality of all the test results. Three months after the HIVST kits application, the index participants completed a follow-up survey to assess their risk behaviors and whether they provided peer support to the alters. More details was shown in (Figure 1).

### Outcomes

The primary outcomes of our study encompassed the proportion of peer support and the occurrence of high-risk behaviors during the HIV self-testing (HIVST) distribution process. We separately delineated index participants who self-reported providing peer support and those who did not provide peer support to the alters. Peer support was defined as index participants providing all of the following four items to the alters: (1) HIV-related knowledge, (2) HIV testing relevant knowledge, (3) how to conduct HIVST, and (4) how to read the results of HIVST. Each item was assessed as a dichotomous response (yes or no). We summed the scores for these four items and created a binary variable indicating whether alters had received comprehensive peer support, covering all assessed aspects. Peer sexual behaviors on the day of HIVST kits distribution were related to the four items, which were also assessed dichotomously: (1) use HIVST at the same time with index participants, (2) had sex with index participants on the day of the test, (3) had sex with index participants before or after the test, and (4) condom use during the sex with index participants. High-risk behaviors were related to the following items: (1) disclosed sexual orientation, (2) had anal sex with men, (3) frequency of condom use during anal sex, (4) condom use during last sexual activity with a male partner, and (5) role in the sex (see Supplementary File). Our analysis included examining factors associated with peer support during the secondary distribution of HIVST among index participants. Additionally, we explored the occurrence of high-risk behaviors between index participants and alters during the HIVST distribution process. Utilizing logistic regression, we analyzed sexual behaviors associated with peer support among alters during the distribution of HIVST. Subsequently, all index participants were subjected to an online 3-month follow-up survey after ordering the kits. We conducted follow-ups with the index participants to assess testing uptake rates and changes in high-risk behaviors among them after ordering the HIVST kits.

### Statistical analysis

All data analyses were conducted in R 4.1.2 for Windows 10. Chi-squared tests were used to compare baseline socio-demographic characteristics of index participants and alters, as well as baseline and follow-up characteristics of index participants’ sexual behaviors. In this study, Cramer’s V was employed to quantify the level of association between the Internet-based HIVST and condomless anal sexual behaviors among index participants. Univariate logistic regression analysis was used to evaluate factors associated with providing peer support among index participants and factors associated with peer support among alter participants. Multivariable logistic regression analysis was conducted to evaluate factors associated with peer support among alter participants. Two-sided *p*-values < 0.05 were considered statistically significant, and odds ratios (OR) with (*95% CI*) were estimated. Covariates, which had a larger number or a wide distribution within the sample, were selected as the reference to ensure the stability and reliability of the model. Factors with a *p*-value of < 0.1 in the univariate analyses for peer support were included in the multivariate logistic regression model.

### Ethical considerations

This study was approved by the Zhuhai Center for Disease Control and Prevention. A signed digital informed consent form was obtained from all the participants before collecting any study information or specimens. Participants joined the study voluntarily and were free to withdraw from the study at any time. The project staff had signed confidentiality agreements with the Zhuhai Center for Disease Control and Prevention.

## Results

### Participants characteristics

Our study recruited a total of 288 participants, and they distributed HIVST kits to 478 alters, who were from 94 cities in China, and most of them were located in Zhuhai. Table 1 shows the characteristics of the study participants. The mean age for index participants and alters was 29.1 (standard deviation [SD], 7.6) years and 27.3 (SD, 7.1) years, respectively. Index participants had higher proportions of obtaining university or higher education than alters (80.9% vs. 71.6%, *p* = 0.004). More index participants had disclosed their same-sex behaviors to individuals than their sexual partners (48.6% vs. 38.3%, *p* = 0.006).

**Table 1:** Characteristics of the index participants and alters.

Variables	Total (*n*, %) (*N* = 766)	Indexes (*n*, %) (*n* = 288)	Alters (*n*, %) (*n* = 478)
Age (Mean ± SD)	28.0 ± 7.4	29.1 ± 7.6	27.3 ± 7.1
Marital status			
Single	635 (82.9)	246 (85.4)	389 (81.4)
Engaged/Married	99 (12.9)	34 (11.8)	65 (13.6)
Separate/Divorced/widowed	32 (4.2)	8 (2.8)	24 (5.0)
Education			
High school and below	191 (24.9)	55 (19.1)	136 (28.5)
College and above	575 (65.1)	233 (80.9)	342 (71.6)
Monthly income (RMB)			
<1,500	72 (9.4)	28 (9.7)	44 (9.2)
1,500 ~ 3,000	81 (10.6)	31 (10.8)	50 (10.5)
3,001 ~ 5,000	199 (26.0)	73 (25.3)	126 (26.4)
5,001 ~ 8,000	234 (30.5)	85 (29.5)	149 (31.2)
>8,000	180 (23.5)	71 (24.7)	109 (22.8)
Sexual orientation			
Homosexual	532 (69.5)	207 (71.9)	325 (68.0)
Bisexual	168 (21.9)	70 (24.3)	98 (20.5)
Others/Uncertain	66 (8.6)	11 (3.8)	55 (11.5)
Disclosed sexual orientation			
Yes	323 (42.2)	140 (48.6)	183 (38.3)
No	443 (57.8)	148 (51.4)	295 (61.7)
Had anal sex with men^#^			
Yes	527 (68.8)	245 (85.1)	282 (59.0)
No	239 (31.2)	43 (14.9)	196 (41.0)
Number of anal sex partners (*n* = 527)			
One	264 (50.1)	103 (42.0)	161 (57.1)
More than one	263 (49.9)	142 (58.0)	121 (42.9)
Frequency of condom use during anal sex^#^ (*n* = 527)			
Every time	301 (57.2)	135 (55.1)	166 (58.9)
Often (more than half)	137 (26.0)	68 (27.8)	69 (24.5)
Occasionally (less than half)	64 (12.1)	29 (11.8)	35 (12.4)
Never	25 (4.7)	13 (5.3)	12 (4.2)
Condom use during last sexual activity with a male partner (*n* = 527)			
Yes	422 (80.1)	200 (81.6)	222 (78.7)
No	105 (19.9)	45 (18.4)	60 (21.3)
Role in the sex^#^ (*n* = 527)			
Receptive	180 (34.2)	69 (28.2)	111 (39.4)
Insertive	222 (42.1)	112 (45.7)	110 (39.0)
Both	125 (23.7)	64 (26.1)	61 (21.6)
Sex with a female partner^#^			
Yes	62 (8.1)	23 (8.0)	39 (8.2)
No	704 (91.9)	265 (92.0)	439 (91.8)
Ever tested for HIV			
Yes	546 (71.3)	263 (91.3)	283 (59.2)
No	220 (28.7)	25 (8.7)	195 (40.8)
Previous method employed for HIV testing (*n* = 546)			
Self-testing	360 (65.9)	177 (61.5)	183 (38.3)
Institutions with HIV related services	186 (34.1)	86 (29.9)	100 (20.9)

^# In the past 3 months.^

Index participants were more likely to have anal sex (85.1% vs. 59.0%, *p* < 0.001) and have more than one anal sex partner in the past 3 months (58.0% vs. 42.9%, *p* < 0.001). The frequency of condom use in the past 3 months and condom use during the last sexual activity were similar between the two groups. Compared with alters, the role during anal sex was more likely to be insertive among index participants in the past 3 months (45.7% vs. 39.0%, *p* = 0.03). Moreover, index participants were more likely to have tested for HIV (91.3% vs. 59.2%, *p* < 0.001).

### Peer-supported HIVST

**Figure 2:**
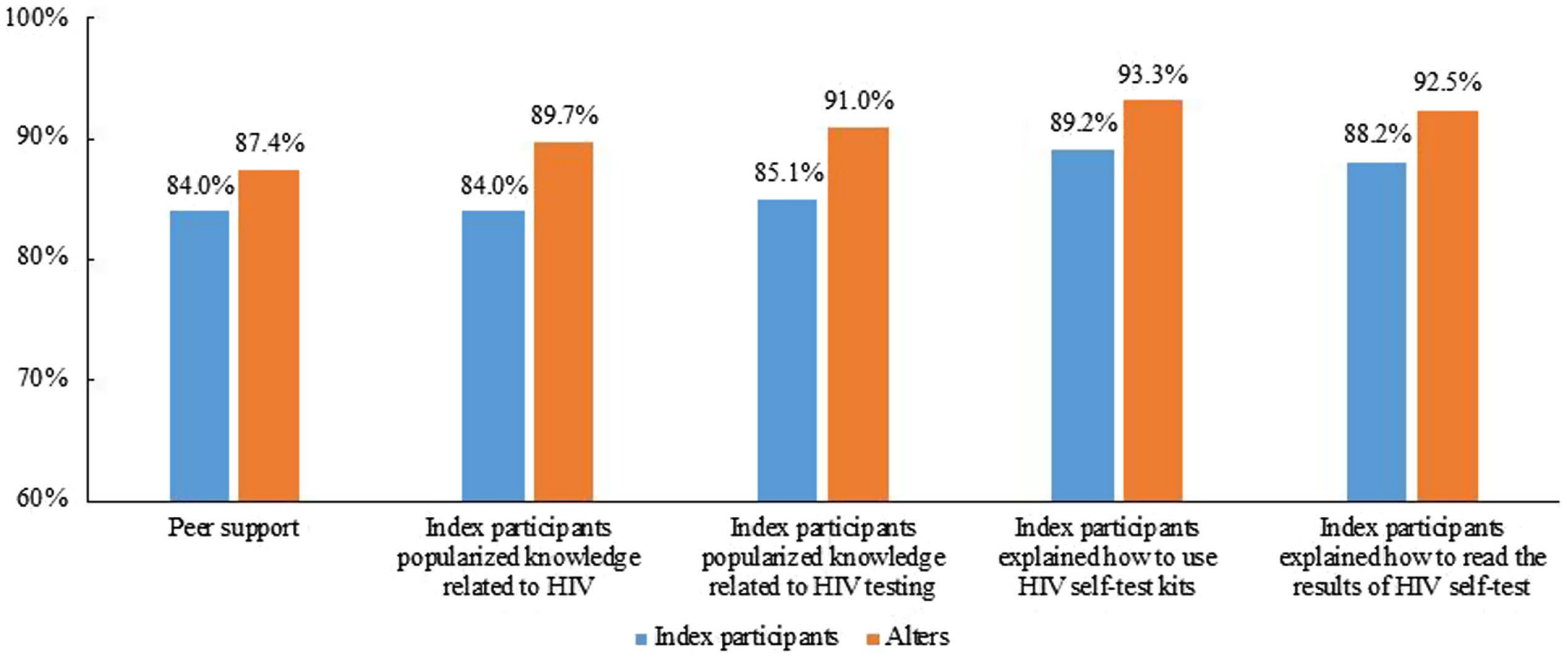
Peer support during secondary distribution of HIVST kits to Index and Alter participants.

Figure 2 shows the peer support during the distribution of HIVST kits. Overall, 84.0% (242/288) of the index participants reported that they provided peer support to the alters and 87.4% (418/478) of the alter participants reported that they received peer support from the index participants.

Overall, 84.0, 85.1, 89.2, and 88.2% index participants reported that they provided HIV-related knowledge, HIV testing relevant knowledge, how to conduct HIVST, and how to read the results of HIVST, respectively, with 89.7, 91.0, 93.3, 92.5% reported by alters.

### Characteristics between index participants who reported providing peer support and not providing peer support

Table 2 shows the characteristics between index participants who reported providing peer support and those not providing peer support among index participants. Index participants ever being asked for advice about HIV and other sexually transmitted infections (STIs) were more likely to provide peer support to alters (odd ratio (20): 1.85 95%CI: 1.01–3.37). There were no significant differences in talking to others about HIV and STIs and providing others with information about HIV and STIs between index participants who reported peer support and non-peer support.

**Table 2:** Factors associated with peer support during the Internet-based HIVST kits secondary distribution among index participants.

Variables	Total (*n* = 288)	Index participants reported peer support (*n* = 232)	Index participants reported non-peer support (*n* = 56)	OR (95%CI)	*p*-value
Talk to others about HIV and STI					
No	57 (19.8)	43 (18.5)	14 (25.0)	Ref	
Yes	231 (80.2)	189 (81.5)	42 (75.0)	1.47 [0.74–2.92]	0.28
Number of people talked about HIV and STI (mean (SD))	1.32 (1.10)	1.37 (1.12)	1.11 (0.97)	1.59 [0.83–3.04]	0.16
Provide others with information about HIV and STI					
No	67 (23.3)	50 (21.6)	17 (30.4)	Ref	
Yes	221 (76.7)	182 (78.4)	39 (69.6)	1.26 [0.95–1.68]	0.12
Possibility of being consulted about HIV and STI					
Unlikely	65 (22.6)	50 (21.6)	15 (26.8)	Ref	
Neutral	186 (64.6)	148 (63.8)	38 (67.9)	1.17 [0.59–2.3]	0.65
Very likely	37 (12.8)	34 (14.6)	3 (5.3)	3.4 [0.91–12.65]	0.07
Ever be asked for advice about HIV and STI					
No	91 (31.6)	67 (28.9)	24 (42.9)	Ref	
Yes	197 (68.4)	165 (71.1)	32 (57.1)	1.85 [1.01–3.37]	0.045*

* *p* < 0.05.

### Sexual behaviors associated with peer support among alters during the distribution of HIVST

Table 3 shows that there were no significant differences in the relationship with index participants, concurrent use of HIVST with index participants, engaging in sexual activity with index participants on the day of testing, having had sex with index participants before or after the test, and condom use during sexual encounters with index participants between alter participants reported peer support and those who did not report peer support.

**Table 3:** Sexual behaviors associated with peer support among alters during the distribution of HIVST kits.

Variables	Total (*n* = 478)	Alters reported peer supports (*n* = 418)	Alters reported non-peer supports (*n* = 60)	OR (95%CI)	*p-*value
The relationship with index participants*					
Friends	258 (54.0)	230 (55.0)	28 (46.7)	Ref	
Regular sex partners	95 (19.9)	81 (19.4)	14 (23.3)	0.70 [0.36, 1.44]	0.32
Casual sex partners	90 (18.8)	75 (17.9)	15 (25.0)	0.61 [0.31, 1.23]	0.15
Other	35 (7.3)	32 (7.7)	3 (5.0)	1.30 [0.43, 5.64]	0.68
Use HIVST at the same time with index participants					
No	259 (54.2)	228 (54.5)	31 (51.7)	Ref	
Yes	219 (45.8)	190 (45.5)	29 (48.3)	0.89 [0.52, 1.54]	0.68
Had sex with index participants on the day of test					
No	383 (80.1)	339 (81.1)	44 (73.3)	Ref	
Yes	95 (19.9)	79 (18.9)	16 (26.7)	0.64 [0.35, 1.22]	0.16
Had sex with index participants before or after the test					
Before the test	72 (75.8)	62 (78.5)	10 (62.5)	Ref	
After the test	23 (24.2)	17 (21.5)	6 (37.5)	0.46 [0.15, 1.51]	0.18
Condom use during the sex with index participants					
Yes	69 (72.6)	58 (73.4)	11 (68.8)		
No	26 (27.4)	21 (26.6)	5 (31.2)	0.80 [0.26, 2.77]	0.70

*: Regular sexual partner: refers to a sexual partner with whom you have a stable relationship. This includes partners in a romantic relationship, such as a spouse, boyfriend, or girlfriend, as well as other sexual partners with whom you maintain a stable relationship (e.g., a regular casual partner). Casual sexual partner: refers to a sexual partner with whom you have maintained a sexual relationship for three months or less. This also includes non-regular casual partners and individuals engaged in commercial sex. Other: refers to all types of relationships that are neither sexual partners nor friends, including family members, colleagues, etc.

Table 4 shows that alters who did not test for HIV were more likely to report peer support, compared with those who had ever taken in HIVST [*aOR:* 2.23, *95%CI*: 1.18–4.37].

**Table 4:** Logistic regression analysis of sexual behaviors associated with peer support among alters during the distribution of HIVST kits.

Variables	Univariate regression analysis		Multivariable regression analysis	
	OR (95%CI)	*p-*value	aOR (95%CI)	*p-*value
Age (Mean ± SD)	1.00 [0.96, 1.04]	0.92	1.01 [0.96, 1.06]	0.73
Marital status				
Single	Ref		Ref	
Engaged/Married	0.97 [0.41, 2.06]	0.95	1.18 [0.42, 3.05]	0.74
Separate/Divorced/widowed	0.98 [0.26, 4.80]	0.98	1.02 [0.25, 5.14]	0.98
Education				
High school and below	Ref		Ref	
College and above	0.59 [0.29, 1.12]	0.12	0.61 [0.29, 1.23]	0.19
Monthly income (RMB)				
3,001 ~ 5,000	Ref		Ref	
1,500 ~ 3,000	1.45 [0.50, 5.31]	0.52	1.44 [0.47, 5.44]	0.55
<1,500	1.67 [0.58, 6.08]	0.38	1.53 [0.52, 5.65]	0.48
5,001 ~ 8,000	0.80 [0.39, 1.57]	0.52	0.90 [0.43, 1.83]	0.77
>8,000	1.07 [0.49, 2.38]	0.86	1.21 [0.52, 2.84]	0.65
Sexual orientation^&^				
Homosexual	Ref			
Bisexual	1.09 [0.57, 2.25]	0.80		
Others/Uncertain	1.52 [0.63, 4.57]	0.40		
Disclosed sexual orientation				
Yes	Ref			
No	1.09 [0.62, 1.88]	0.77		
Had anal sex with men^#^				
Yes	Ref			
No	1.23 [0.71, 2.19]	0.47		
Frequency of condom use during anal sex^#^				
Every time	Ref			
Often (more than half)	1.72 [0.75, 4.48]	0.23		
Occasionally (less than half)	3.21 [0.90, 20.49]	0.12		
Never	0.97 [0.24, 6.55]	0.97		
Condom use during last sexual activity with a male partner				
Yes	Ref			
No	1.02 [0.46, 2.50]	0.97		
Role in the sex^#^				
Receptive	Ref			
Insertive	0.61 [0.29, 1.27]	0.19		
Both	2.79 [0.87, 12.47]	0.12		
No	1.14 [0.55, 2.31]	0.72		
Sex with a female partner^#^				
No	Ref			
Yes	1.28 [0.49, 4.40]	0.65		
Previous method employed for HIV testing				
Self-testing	Ref		Ref	
Institutions with HIV related services	1.55 [0.78, 3.28]	0.23	1.54 [0.77, 3.28]	0.24
Never	2.37 [1.27, 4.59]	0.008*	2.23 [1.18, 4.37]	0.015*

^#^: In the past 3 months. *: *p* < 0.05. ^**&**^: The participants’ self-identified sexual orientation, where “other/uncertain” refers to individuals who do not identify as either homosexual or bisexual (Heterosexual individuals were excluded).

### Changes in sexual behaviors in index participants

Table 5 shows the changes in sexual behaviors in index participants after 3 months of follow-up. After 3 months of follow-up, there was a moderate level of association between Internet-based HIVST and a decrease in the occurrence of anal sex with men, as indicated by a Cramer’s V coefficient of 0.20. However, there was no significant difference between the Internet-based HIVST and condomless anal sexual behaviors among index participants.

**Table 5:** Changes in sexual behaviors among index participants after 3-month follow-up.

Variables	Baselin (*n* = 288, %)	Follow up (*n* = 288, %)	*p-*value	Cramer’s *V* value
Number of men whom you had anal sex with in the past 3 months			<0.001*	0.20
0	43 (14.9)	88 (30.5)		
1	103 (35.8)	99 (34.4)		
≥2	142 (49.3)	101 (35.1)		
Condom use during anal sex with men			0.60	0.06
Consistently	135 (55.1)	111 (55.5)		
Often (more than half)	68 (27.8)	47 (23.5)		
Occasionally (less than half)	29 (11.8)	27 (13.5)		
Never	13 (5.3)	15 (7.5)		

*: *p* < 0.05.

## Discussion

Internet/digital-based HIVST among MSM has demonstrated a very high rate of usage in China (21). Understanding whether the index MSM will provide support to the alters is essential in planning network distribution of HIVST kits to improve HIV testing and intervention coverage. Our study contributes to the literature by assessing the occurrence of peer support and HIV-related high-risk behaviors during the process of HIVST kits distribution among MSM in China. We found that the proportion of index participants conducting peer support during the distribution of self-testing kits was over 84%, a very high level, which is an important intervention measure for alter participants. Meanwhile, after 3 months of follow-up, there was a moderate level of association between Internet-based HIVST and a decrease in the occurrence of anal sex with men, indicating that self-testing can influence the occurrence of high-risk behaviors. This study has significant public health implications for the promotion of peer support among MSM through self-testing kits distribution, as well as the simultaneous reduction of high-risk behaviors through both testing and peer support.

Our study demonstrated three important benefits over previously studied online secondary distribution of HIVST (14, 22). First, our study showed that peer support occurred during the process of Internet-based HIVST kits distribution and maintained a high frequency. Not only did the index participants take the initiative to provide peer support to the alters but also the alters self-reported receiving peer support from the index participants. This is a crucial finding, as it suggests that the digital-based peer support within the MSM community potentially enhances the effectiveness of HIV prevention initiatives. Peer support could advance the awareness of HIV testing and provide information about the location and availability of HIV services, and offer social support and referrals for services, which increase access to facility-based HIV testing services and improve HIV testing uptake (23, 24). As the main part of peer support, HIV-related knowledge delivery to the key population will promote HIV prevention (3). Our previous study demonstrated that alters have a significantly higher HIV infection rate than index participants (14); this implies that, as a group of MSM, alters should be the focus of targeted interventions, and Internet-based HIVST kits distribution is an important approach to provide peer support to a group of MSM who lack HIV-related prevention measures and may hold a strong promise to improve peer support coverage. While a meta-analysis revealed that HIVST was linked to a 47% lower rate of linkage to care compared to facility-based HIV testing, this disparity was attributed to insufficient counseling support in self-testing contexts (25).

Second, we observed that the index participants who delivered HIVST kits were significantly reduced for having anal sex and number of sex partners; meanwhile, the results of having condom sex during the 3 months follow-up were significantly increased. Reducing risk behaviors such as these would result in new HIV infections declination. In the United States, HIV incidence has declined overall in recent years, but rates of new infections remain high, particularly among MSM (26). Nearly all of these new infections occur as a direct result of sexual risk behavior, which among MSM involves having insertive or receptive anal sex without using an effective method of prevention (27, 28). A meta-analysis of 15 studies also found that peer-led HIV interventions might reduce unprotected anal intercourse among HIV-negative MSM (7). Meanwhile, a great deal of evidence shows that the Internet has emerged as the most popular platform for facilitating sex networking among MSM (29–33). Another study in Hong Kong found that Internet-based interventions have used several different aspects, including disseminating prevention messages (34). These findings suggested that internet-based HIVST appears to be a promising approach of behavior interventions among MSM.

Third, we found that risk behaviors occurred in the process of HIVST kits distribution between the index and the alters, such as the index participants engaging in condomless sex with alters and engaging in sex behaviors before HIV testing. These risk behaviors can result in HIV transmission. Joint United Nations Program on HIV/AIDS (UNAIDS) proposed the “95-95-95” target, which increases the capacity for identification of new cases for early diagnosis and immediate treatment, helping to control the spread of the virus (35–37). Increasing awareness of HIV status can decrease risk behaviors in key populations. Engaging in sexual activity before HIV testing may hinder the immediate detection of potential HIV infections and impede efforts to halt HIV transmission. Even though the self-testing results are negative, we still suggest practicing safe sex with condoms due to the existence of window periods. Therefore, providing additional warning information on high-risk behaviors and the window period may be helpful in reducing such behaviors.

Our study has limitations. First, our study was an integration of our previous two studies from which we figured out the data of peer support and risk behaviors (14, 38). Therefore, we are not sure the sample size is satisfied for our purpose. However, it could be an important reference for further study in this area. Second, we have selection bias in our study, and we measured index participants based on those who delivered HIVST kits and analyzed their demographic characteristics. However, in reality, the absence of index participants to deliver HIVST does not necessarily indicate a lack of capability to deliver HIVST kits to alters. Therefore, information about this subset of individuals is missing from the sample. Third, we did not make follow-ups for the alters, so we do not know whether the risk behaviors were reduced for alters. In our previous studies, we demonstrated that alters are the primary users of HIVST kits, but we believe that if they apply the HIVST kits themselves or make a secondary distribution, then the alters will become the index, and their risk behaviors will be changed similar to the index participants in this study (14, 38). Fourth, this study did not ask the participants about the site where the peer support occurred, whether it happened online by chatting APP such as WeChat or in person, and which was the main situation. Finally, our study relies on self-reported data, introducing potential recall and social desirability biases.

## Conclusion

This study has enhanced our understanding of the occurrence of peer support and HIV-related high-risk behaviors during the process of HIVST kits secondary distribution. These findings have important implications for improving HIV prevention coverage and have the potential to reduce risk behaviors among key populations. This study provides evidence that Internet-based HIVST appears to be a promising approach for behavior interventions among MSM.

## Data Availability

The data analyzed in this study is subject to the following licenses/restrictions: the data sets generated and analyzed during this study are not publicly available due confidentiality principle. Requests to access these datasets should be directed to 930224lh@163.com.
