# Supplementary material for: Peer support to improve the secondary distribution of Internet-based HIV self-testing kits among men who have sex with men in Zhuhai, China
Source: Front Public Health. 2025 Apr 29;13:1522425. doi: 10.3389/fpubh.2025.1522425 (PMC12069058; doi:10.3389/fpubh.2025.1522425)
Supplement: Supplementary file 1 [file Supplementary_file_1.docx]

**Questionnaire I (Baseline questionnaire)**

1. [Number] What is your age?  

______years old （If you are younger than 16 years old, you do not meet the requirements and are excluded from the questionnaire）

1. [Single choice] What is your sex at birth? (Biological sex)

- Male
- Female（Do not meet the requirements, excluded from the questionnaire）

1. [Single choice] Have you ever had sexual intercourse with a man?

- Yes
- No（Do not meet the requirements, excluded from the questionnaire）

1. [Single choice] Are you willing to complete our follow-up survey in 3 months?

- Willing
- Not willing（Do not meet the requirements, excluded from the questionnaire）

## A. Sociodemographic information

1. [Single choice]What is your marital status?

- Engaged/married
- Unmarried
- Separated/divorced
- Widowed

1. [Single choice] What is your household registration?

- Rural in other provinces
- Registered in the city
- Other city in this province
- Rural in this province
- Urban in other provinces

1. [Single choice]What is your ethnicity?

- Han
- Other, (Please add which ethnic group)

1. [Single choice]Your level of education is?

- high school or less
- bachelor's degree
- PhD, Master's or above

1. [Single choice] What is your employment status?

- student
- public service
- farmer
- laborer (blue collar)
- office worker (white collar)
- service
- technician
- sex worker
- Unemployed
- other：______

1. [Single choice]What is your average monthly income?

- Less than 1500 yuan
- 1500－3000 yuan
- 3001-5000 yuan
- 5001-8000 yuan
- More than 8000 yuan

1. [Single choice]What is your gender identity?

- male
- female
- transgender
- other/unsure

1. [Single choice] What is your sexual orientation?

- gay
- heterosexual
- bisexual
- unsure

1. [Single choice]Have you disclosed your sexual orientation or sexual activity with men to others? (including health workers)

- Yes
- No

## B. Male sex information(Q)

1. [Single choice] Have you had anal sexual intercourse with a man in the last 6 months?

- Yes
- No **[Skip to problem C]**

1. [Number] In the last 6 months, with how many men have you had anal sexual intercourse?

     The exact number of people is very important for scientific statistics, please carefully recall and fill in.

1. [Multiple choice] In the last 6 months, where did you look for male sexual partners?

- bars, dance halls, tea rooms, meetings
- saunas, massage parlors
- parks, public spaces, on the streets
- online forums
- social media (e.g. Blued, Jackd, QQ)
- through friends
- other     (Please add clarification)
- did not seek

1. [Single choice] In the last six months, what was your role when you had sex with men?

- insertive ("1")
- receptive ("0")
- both

1. [Single choice]In the last six months, how frequently did you use condoms when you had anal sexual intercourse with men?

- never
- rarely (less than half the time)
- frequently (more than half the time)
- every time

1. [Single choice]Did you use a condom the most recent time you had anal sex with a man?

- Yes
- No

1. [Single choice]In the last 6 months, how many stable male partners did you have?

Stable male partners are sexual partners who have stable relationships, including boyfriends (BF) who establish romantic relationships and other same-sex partners who maintain stable relationships.

- 0 **[Skip to problem22]**
- other，____

1. [Single choice]In the last 6 months, how frequently did you use condoms when you had sex with stable male partners?

- never
- rarely (less than half the time)
- frequently (more than half the time)
- every time

1. [Single choice]In the last 6 months, how many casual male partners did you have?

Casual male partners: Men with whom you have a sexual relationship of less than 3 months or equal. This also includes casual sex partners and commercial sex partners.

- 0  **[Skip to problem C]**
- other，____

1. [Single choice]In the last 6 months, how frequently did you use condoms when you had sex with casual male partners?

- never
- rarely (less than half the time)
- frequently (more than half the time)
- every time

## C. Sexual behavior with women (Q)

1. [Single choice]In the last 6 months, did you have sex with women?

- Yes
- No **[Skip to problem D]**

1. [Number]With how many different women did you have sex in the last 6 months?

     The exact number of people is very important for scientific statistics, please carefully recall and fill in.

1. [Single choice]In the last 6 months, how frequent did you use condoms when you had sex with women?

- never
- rarely (less than half the time)
- frequently (more than half the time)
- every time

1. [Single choice]Did you use a condom when you most recently had sex with a woman?

- Yes
- No

1. [Single choice]In the last 6 months, how many stable female sexual partners did you have?

(Stable female sexual partners are sexual partners who have stable relationships, including girlfriends who establish romantic relationships and other female sexual partners who maintain stable relationships.)

- 0  **[Skip to problem 30]**
- Other, ____

1. [Single choice]In the last 6 months, how frequent did you use condoms when you had sex with stable female partners?

- never
- rarely (less than half the time)
- frequently (more than half the time)
- every time

1. [Single choice]In the last 6 months, how many casual female sexual partners did you have?

(Casual female partners: women with whom you have a sexual relationship of less than 3 months or equal. This also includes casual sex friends and commercial sex partners.)

- 0 **[Skip to problem D]**
- other，____

1. [Single choice]How frequently do you use condoms when you have sex with casual female sexual partners?

- never
- rarely (less than half the time)
- frequently (more than half the time)
- every time

## D. Influence within one's social circle

1. [Single choice]Overall, how frequently do you discuss HIV/STI related topics with others (including on- and off-line)？

1 2 3 4 5

Never（**Skip to problem E**） very often

1. [Single choice]When you discuss HIV/STI related topics with others, how information do you provide them (including on- and off-line)?

1 2 3 4 5

almost nothing a lot

1. [Single choice]In the last 3 months, with how many people did you discuss HIV/STI related topics (including on- and off-line)?

1 2 3 4 5

none a lot of people

1. [Single choice]How likely are others in your social network to seek advice from you about HIV/STIs (including on- and off-line)?

1 2 3 4 5

very unlikely very likely

1. [Single choice]When you discuss HIV/STI related topics with others in your network, what of the following is the most likely scenario? (including on- and off-line)

1 2 3 4 5

others always give you information you always give others information

1. [Single choice] Overall, when you discuss HIV/STIs with others, how frequently do they seek advice from you (on-or off-line)?

1 2 3 4 5

Never very often

## E. HIV test and HIV self-test kits application

1. [Single choice] Have you ever tested for HIV before this time?

- Yes
- No **[Skip to problem 43]**

1. [Single choice] How did you test for HIV the previous time?

- self test
- Hospital/Community Health Service Center**[Skip to problem 41]**
- Gay Men's Organization（e.g. Xutong）**[Skip to problem 41]**
- Blood donation Agency**[Skip to problem 41]**
- Centers for Disease Control and Prevention**[Skip to problem 41]**

1. [Single choice] How did you obtain your previous HIV self-test kit?

- purchased from an online platform (e.g. Taobao or MSM online services)
- gift from a friend
- purchased from a pharmacy
- through CDC or clinic
- MSM offline services
- other       (Please add clarification)

1. [Single choice] When was the last time you tested for HIV?

- <3 months
- 3-6 months
- >6 months

1. [Single choice]Do you know your last test result?

- Yes, negative
- Yes, positive
- Don't know

1. [Single choice]How many self test kits do you want to request this time? (*One person can apply for a maximum of 5, each deposit 100 yuan, within 3 months according to the number of feedback results refund deposit, such as feedback 1 result refund 100 yuan, 5 copies of 500 yuan*)

- 1
- 2
- 3
- 4
- 5

1. What is your phone number? [**System sends verification code**]
2. Please enter the verification code:

This is the end of the questionnaire

Thank you for using our free HIV self-test kits service.
