# Supplementary material for: Peer support to improve the secondary distribution of Internet-based HIV self-testing kits among men who have sex with men in Zhuhai, China
Source: Front Public Health. 2025 Apr 29;13:1522425. doi: 10.3389/fpubh.2025.1522425 (PMC12069058; doi:10.3389/fpubh.2025.1522425)
Supplement: Supplementary file 2 [file Supplementary_file_2.docx]

**3 month For Index at follow up**

1. What phone number did you use to apply for HIV self-test kits last time?
2. [Single choice] How many kits did you request? ______

- 1
- 2
- 3
- 4
- 5

1. [Single choice] How many kits did you successfully distribute？

- 0 **[run to question 6]**
- Other, （fill a number）

***If choose other，Please answer the following questions about the recipient in chronological order.（Please answer the questions related to all the recipients based on the number of recipients.）***

| Alter | Gender | Relationship to Index | Did you test at the same time with this person? (partner testing) | Recipient's HIV test result is? | If this recipient is your sexual partner or a hookup, did you have sex on the day that the recipient performed the self-test? | If you had sex with this person, was it before or after the self-test?(it is ok to no answer) | If you had sex with this person, did you use a condom? |
| --- | --- | --- | --- | --- | --- | --- | --- |
| First recipient | 1. male  2. female | 1.stable sexual partner  2.casual sexual partner  3.friends  4.family  5.other | 1. yes  2. no | 1.positive  2.negative  3.don’t know | 1. yes  2. no  3. This recipient is not my sexual partner or a hookup | 1.before self-test  2.after self-test  3. this recipient is not my sexual partner or a hookup | 1. yes  2. no  3. This recipient is not my sexual partner or a hookup |
| Second recipient | 1. male  2. female | 1.stable sexual partner  2.casual sexual partner  3.friends  4.family  5.other | 1. yes  2. no | 1.positive  2.negative  3.don’t know | 1. yes  2. no  3. This recipient is not my sexual partner or a hookup | 1.before self-test  2.after self-test  3. this recipient is not my sexual partner or a hookup | 1. yes  2. no  3. This recipient is not my sexual partner or a hookup |
| Third recipient | 1. male  2. female | 1.stable sexual partner  2.casual sexual partner  3.friends  4.family  5.other | 1. yes  2. no | 1.positive  2.negative  3.don’t know | 1. yes  2. no  3. This recipient is not my sexual partner or a hookup | 1.before self-test  2.after self-test  3. this recipient is not my sexual partner or a hookup | 1. yes  2. no  3. This recipient is not my sexual partner or a hookup |
| Fourth recipient | 1. male  2. female | 1.stable sexual partner  2.casual sexual partner  3.friends  4.family  5.other | 1. yes  2. no | 1.positive  2.negative  3.don’t know | 1. yes  2. no  3. This recipient is not my sexual partner or a hookup | 1.before self-test  2.after self-test  3. this recipient is not my sexual partner or a hookup | 1. yes  2. no  3. This recipient is not my sexual partner or a hookup |
| Fifth recipient | 1. male  2. female | 1.stable sexual partner  2.casual sexual partner  3.friends  4.family  5.other | 1. yes  2. no | 1.positive  2.negative  3.don’t know | 1. yes  2. no  3. This recipient is not my sexual partner or a hookup | 1.before self-test  2.after self-test  3. this recipient is not my sexual partner or a hookup | 1. yes  2. no  3. This recipient is not my sexual partner or a hookup |

Regular sexual partner: refers to a sexual partner with whom you have a stable relationship. This includes partners in a romantic relationship, such as a spouse, boyfriend, or girlfriend, as well as other sexual partners with whom you maintain a stable relationship (e.g., a regular casual partner).

Casual sexual partner: refers to a sexual partner with whom you have maintained a sexual relationship for three months or less. This also includes non-regular casual partners and individuals engaged in commercial sex.

1. [Single choice] Consider the most important or closest recipient to you. Did you do the following activities when delivering a kit to this person?

| Share knowledge regarding HIV | *[1]* Yes *[2]* No |
| --- | --- |
| Share knowledge regarding HIV testing | *[1]* Yes *[2]* No |
| Explain the use of HIV self-testing kits | *[1]* Yes *[2]* No |
| Explain how to interpret results of HIV self-testing | *[1]* Yes *[2]* No |
| The recipient did not need me to explain because he/she is very familiar with the above knowledge | *[1]* Yes *[2]* No |
| Were you present when this person tested? | *[1]* Yes *[2]* No |

1. [Single choice] Consider the most important or closest recipient to you. Did you experience any of the following when delivering a kit to this person?

| The recipient didn't understand why you gave him/her a self-test kit | *[1]* Yes *[2]* No |
| --- | --- |
| The recipient felt ashamed because you gave him/her a self-test kit | *[1]* Yes *[2]* No |
| The recipient felt you do trust him/her because you gave him/her a self-test kit | *[1]* Yes *[2]* No |
| The recipient had verbal conflict with you, such as arguing or verbally abusing you | *[1]* Yes *[2]* No |
| The recipient had physical conflict with you, such as forceful pushing or pulling or hitting | *[1]* Yes *[2]* No |
| You used forceful methods, such as threatening him/her, treating him/her coldly, or using violence, to make the recipient accept testing | *[1]* Yes *[2]* No |
| The recipient's relationship with you became distant because you requested that he/she perform testing | *[1]* Yes *[2]* No |

1. [Single choice] How many self-testing kits did you use on yourself?

- 0 **[run to question 16]**
- Other, （number）

1. [Single choice] Was someone else present with you during your most recent self-test?

- Yes
- No

1. [Single choice]What is the most important reason that encourages you to self-test?

- convenient, saves time
- accurate
- protects my privacy
- easy to use
- I don't want to go to clinic to test
- other (all of the above)

1. [Single choice] The result of your most recent self-test is?

- positive
- negative  **[run to question 16]**
- don't know/don't want to say  **[run to question 16]**

1. [Single choice] Did you go to a health center for a confirmation test?

- Yes
- No
- don't know/don't want to say

*“Confirmatory testing” refers to conducting another round of testing after obtaining the preliminary HIV test results to confirm the final outcome.*

1. [Single choice] Have you been to a health center to seek treatment services?

- Yes
- No
- don't know/don't want to say

1. [Single choice]Since you last tested, with about how many men have you had anal sex?

- 0 **[run to question 14]**
- Other,___（number）

1. [Single choice] Since you last tested, how often do you wear a condom when you have anal sex with men?

- never
- rarely (less than half the time)
- frequently (more than half the time)
- every time

1. [Single choice] Since you last tested, with about how many women have you had sex?

- 0 **[run to question 16]**
- Other,___（number）

1. [Single choice] Since you last tested, how often do you wear a condom when you have sex with women?

- never
- rarely (less than half the time)
- frequently (more than half the time)
- every time

1. [Single choice] If both self-testing and testing in a clinic are both free, which method of testing would you choose?

- HIV self-test
- testing in a clinic
- the same
- unsure

1. [Single choice]How frequently do you plan to test for HIV？

- 0=once every 3 months or more frequently
- once every 3-6months
- once every 6-12months
- once every month or less frequently
- don't plan to test again
- don't know

**The end**
