# Supplementary material for: Peer support to improve the secondary distribution of Internet-based HIV self-testing kits among men who have sex with men in Zhuhai, China
Source: Front Public Health. 2025 Apr 29;13:1522425. doi: 10.3389/fpubh.2025.1522425 (PMC12069058; doi:10.3389/fpubh.2025.1522425)
Supplement: Supplementary file 3 [file Supplementary_file_3.docx]

**Questionnaire III [For HIV self-testing kit recipients]**

1. [Single choice] What is your relationship to the index participant?

- stable same-sex sexual partner
- casual same-sex sexual partner
- stable heterosexual partner
- casual heterosexual partner
- gay friend
- straight friend
- family member
- other

Permanent partners are sexual partners who have stable relationships, including marriage, male/girlfriend and other sexual partners who maintain stable relationships (regular friends with benefits).

A casual partner is a sexual partner who has maintained a sexual relationship with you for less than or equal to 3 months, which also includes non-regular friends and commercial service.

1. [Number] What is your age? μ______years old **（If you are younger than 16 years old, you do not meet the requirements and are excluded from the questionnaire）**
2. [Single choice] What was your sex at birth? (Biological sex)
   - - male
     - female **[Skip to problem C]**
3. [Single choice] What is your gender identity?
   - - male
     - female
     - Transgender
     - other/unsure
4. [Single choice] What is your sexual orientation?
   - - gay
     - Heterosexual
     - bisexual
     - unsure
5. [Single choice] Have you disclosed your sexual orientation or sexual activity with men to others? (including health workers)
   - - Yes
     - No

## A．Sexual behavior with male

1. [Single choice] Have you had anal sexual intercourse with a man in the last 6 months?
   - - Yes
     - No **[Skip to problem B]**
2. [Number] In the last 6 months, with how many men have you had anal sexual intercourse?

     The exact number of people is very important for scientific statistics, please carefully recall and fill in.

1. [Multiple choice] In the last 6 months, where did you look for male sexual partners?

- bars, dance halls, tea rooms, meetings
- saunas, massage parlors
- parks, public spaces, on the streets
- online forums 
- social media (e.g. Blued, Jackd, QQ)
- through friends
- other
- did not seek

1. [Single choice] In the last 6 months, what was your role when you had sex with men?

- insertive ("1")
- Receptive ("0")
- both

1. [Single choice] In the last 6 months, how frequently did you use condoms when you had anal sexual intercourse with men?

- never
- rarely (less than half the time)
- frequently (more than half the time)
- every time

1. [Single choice] Did you use a condom the most recent time you had anal sex with a man?

- Yes
- No

1. [Single choice] In the last 6 months, how many stable male partners did you have?

stable sexual partner refers to a sexual companion with a stable relationship, including a boyfriend (BF) in a romantic relationship and other same-sex sexual partners who maintain a stable relationship (regular casual sex partner/4N9)

- - - 0  **[Skip to problem15]**
    - Other,____

1. [Single choice] In the last 6 months, how frequently did you use condoms when you had sex with stable male partners?

- never
- rarely (less than half the time)
- frequently (more than half the time)
- every time

1. [Single choice] In the last 6 months, how many casual male partners did you have?

Casual male partners: Men with whom you have a sexual relationship of less than 3 months or equal. This also includes casual sex partners and commercial sex partners.

- 0人 **[Skip to problem B]**
- Other,____

1. [Single choice] In the last 6 months, how frequently did you use condoms when you had sex with casual male partners?

- never
- rarely (less than half the time)
- frequently (more than half the time)
- every time

## B．Sexual behavior with women

1. [Single choice] In the last 6 months, did you have sex with women?

- Yes
- No **[Skip to problem C]**

1. [Number] With how many different women did you have sex in the last 6 months?

     The exact number of people is very important for scientific statistics, please carefully recall and fill in.

1. [Single choice] In the last 6 months, how frequent did you use condoms when you had sex with women?

- never
- rarely (less than half the time)
- frequently (more than half the time)
- every time

1. [Single choice] Did you use a condom when you most recently had sex with a woman?

- Yes
- No

1. [Single choice] In the last 6 months, how many stable female sexual partners did you have?

Permanent partners are sexual partners who have stable relationships, including girlfriends who establish romantic relationships and other female sexual partners who maintain stable relationships (regular friends with benefits).

- 0  **[Skip to problem 23]**
- Other,____

1. [Single choice] In the last 6 months, how frequent did you use condoms when you had sex with stable female partners?

- never
- rarely (less than half the time)
- frequently (more than half the time)
- every time

1. [Single choice] In the last 6 months, how many casual female sexual partners did you have?

Casual female partners: women with whom you have a sexual relationship of less than 3 months or equal. This also includes casual sex friends and commercial sex partners.

- 0  **[Skip to problem C]**
- Other,____

1. [Single choice]How frequently do you use condoms when you have sex with casual female sexual partners?

- never
- rarely (less than half the time)
- frequently (more than half the time)
- every time

## C． Self-test kits received and testing experience

1. [Single choice]Regarding the following questions, did your index do the following when he gave you the self-testing kit?

| shared knowledge regarding HIV with me | *[1]* Yes *[2]* No |
| --- | --- |
| shared knowledge regarding HIV testing with me | *[1]* Yes *[2]* No |
| explained the use of HIV self-testing kits to me | *[1]* Yes *[2]* No |
| explained how to interpret results of HIV self-testing to me | *[1]* Yes *[2]* No |
| I did not need the index partner to explain anything because I am very familiar with all of the above | *[1]* Yes *[2]* No |
| When I tested, the index partner was with me | *[1]* Yes *[2]* No |

1. [Single choice]Did you experience any of the following during the process of receiving the self-test kit from your partner?

| I did not understand at the time why he gave me a self-test kit | *[1]* Yes *[2]* No |
| --- | --- |
| I felt that his giving me a kit was to shame me | *[1]* Yes *[2]* No |
| I felt that he does not trust me | *[1]* Yes *[2]* No |
| Because he gave me the self-test kit, we had some verbal conflict, such as arguing, verbal abuse, etc. | *[1]* Yes *[2]* No |
| Because he gave me the self-test kit, we had some physical conflict, such as forceful pushing or hitting etc. | *[1]*A Yes *[2]* No |
| My partner used forceful methods to make me accept the test, including threats, treating me coldly, or using force | *[1]* Yes *[2]* No |
| Because my partner made me test, our relationship has become distant | *[1]* Yes *[2]* No |

1. How did this self-test experience affect you?

| Compared to before, my knowledge and awareness of HIV/AIDS is: | *[1]* improved *[2]* the same *[3]* worse |
| --- | --- |
| Compared to before, my knowledge and awareness of HIV/AIDS testing is: | *[1]* improved *[2]* the same *[3]* worse |
| Compared to before, my acceptance of HIV testing is: | *[1]* improved *[2]* the same *[3]* worse |
| Compared to before, my acceptance of condomless sex is: | *[1]*more acceptable *[2]*the same*[3]*less acceptable |

1. [Single choice]How soon did you use the test kit after receiving it?__________(Enter the year month day)
2. [Single choice]How do you personally feel about the ease of using this self-testing kit?

- very easy
- relatively easy
- relatively hard
- very hard

1. [Single choice]Did you test for HIV at the same time as the index? (partner testing)

- Yes
- No **[Skip to problem 32]**

1. [Single choice]Do you know the test result of the index partner?

- Yes, positive
- Yes, negative
- No, I don't know his test result

1. [Single choice]Does the index partner know your result?

- Yes, he knows I'm positive
- Yes, he knows I'm negative
- No, he doesn't know my test result

1. [Single choice]On the day that you tested, did you have sex with the index partner?

- Yes
- No **[Skip to problem 36]**

1. [Single choice] Did you have sex before or after the test?

- Before
- After

1. [Single choice]When you had sex with your index partner, did you use a condom?

- Yes
- No

1. [Single choice]Have you ever tested for HIV before this time?

- Yes
- No **[Skip to problem 40]**

1. [Single choice] How did you test for HIV the previous time?

- HIV self-test
- Hospital/Community Health Service Center**[Skip to problem 39]**
- Gay Men's Organization（e.g. Xutong）**[Skip to problem 39]**
- Blood donation Agency**[Skip to problem 39]**
- Centers for Disease Control and Prevention**[Skip to problem 39]**

1. [Single choice] How did you obtain your previous HIV self-test kit?

- purchased from an online platform (e.g. Taobao or MSM online services)
- gift from a friend
- purchased from a pharmacy
- through CDC or clinic
- MSM offline services
- other

1. [Single choice] Do you know the result of your prior test?

- Yes, positive
- Yes, negative
- Don't know

1. [Single choice] What was your test result this time?

- Positive
- Negative **[Skip to problem 48]**
- Don't know/don't want to share **[Skip to problem 48]**

1. [Single choice] Did you go to a health center for confirmation testing?

- Yes
- No
- Don't know/don't want to share

*"Confirmatory testing" means that after the initial HIV test results are obtained, another round of testing is performed to confirm the final result.*

1. [Single choice] Did you go to a health center for medical services?

- Yes
- No
- don't know/don't want to share

1. [Single choice] Have you previously used medicine to prevent HIV (PrEP)?

- Yes
- No

1. [Single choice] From the day that you tested until today, with how many men did you have anal sexual intercourse?

- 0 **[Skip to problem 46]**
- Other,_________

1. [Single choice] Since you tested until today, how often did you use a condom when you had anal sex with men?

- never
- rarely (less than half the time)
- frequently (more than half the time)
- every time

1. [Single choice] Since you tested until today, with how many women did you have sexual intercourse?

- 0 **[Skip to problem 48]**
- Other,_________

1. [Single choice] Since you tested until today, how often did you use a condom when you had sex with women?

- never
- rarely (less than half the time)
- frequently (more than half the time)
- every time

1. [Single choice] If both self-testing and testing in a clinic are both free, which method of testing would you choose?

- HIV self-test
- testing in a clinic
- the same
- unsure

1. [Single choice] How frequently do you plan to test for HIV?

- once every 3 months or more frequently
- once every 3-6months
- once every 6-12months
- once every 12 months or less frequently
- I don't plan to test again
- Don't know

## D． Sociodemographic information

1. [Single choice] What is your marital status?

- Engaged/married
- Unmarried
- Separated/divorced
- Widowed

1. [Single choice]What is your household registration?

- Registered in the city
- Other city in this province
- Rural in this province
- Urban in other provinces
- Rural in other provinces

1. [Single choice] What is your ethnicity?

- Han
- Other

1. [Single choice] Your level of education is?

- High school or less
- technical school
- bachelor's degree
- PhD, Master's or above

1. [Single choice] What is your employment status?

- student
- public service
- farmer
- laborer (blue collar)
- office worker (white collar)
- service
- technician
- sex worker
- unemployed
- Other,______

1. [Single choice] What is your average monthly income?

- Less than1500
- 1500－3000
- 3001-5000
- 5001-8000
- More than 8000

1. What's your mobile number? **[System sends verification code]**
2. Please enter the verification code:

This is the end of the questionnaire

Thank you for using our free HIV self-test kits service.
